# Strontium-Substituted Bioceramics Particles: A New Way to Modulate MCP-1 and Gro-α Production by Human Primary Osteoblastic Cells

**DOI:** 10.3390/ma9120985

**Published:** 2016-12-05

**Authors:** Julien Braux, Frédéric Velard, Christine Guillaume, Marie-Laure Jourdain, Sophie C. Gangloff, Edouard Jallot, Jean-Marie Nedelec, Patrice Laquerrière, Dominique Laurent-Maquin

**Affiliations:** 1 EA 4691 Biomatériaux et Inflammation en Site Osseux (BIOS), SFR CAP-Santé (FED 4231), University of Reims-Champagne-Ardenne, 1 Avenue du Maréchal Juin, 51095 Reims CEDEX, France; frederic.velard@univ-reims.fr (F.V.); christine.guillaume@univ-reims.fr (C.G.); marie-laure.jourdain@etudiant.univ-reims.fr (M.-L.J.); sophie.gangloff@univ-reims.fr (S.C.G.); dominique.laurent-maquin@univ-reims.fr (D.L.-M.); 2 UFR Odontologie, University of Reims Champagne Ardenne, 2 rue du Général Koenig, 51100 Reims, France; 3 University Hospital of Reims, 45 rue Cognacq Jay, 51100 Reims, France; 4 UFR de Pharmacie, University of Reims Champagne Ardenne, 1 Avenue du Maréchal Juin, 51095 Reims CEDEX, France; 5 Laboratoire de Physique Corpusculaire de Clermont-Ferrand, UMR 6533, CNRS/IN2P3, Université Clermont Auvergne, 24 Avenue des Landais, 63177 Aubiere CEDEX, France; Edouard.JALLOT@univ-bpclermont.fr; 6 Institut de Chimie de Clermont-Ferrand (ICCF), UMR 6296, CNRS, Université Clermont Auvergne, SIGMA Clermont, F-63000 Clermont-Ferrand, France; jean-marie.nedelec@sigma-clermont.fr; 7 Institut Pluridisciplinaire Hubert Curien (IPHC), UMR 7178, CNRS, Université de Strasbourg, 23 rue de Loess, F-67000 Strasbourg, France; patrice.laquerriere@iphc.cnrs.fr

**Keywords:** human primary bone cells, cytokines, strontium, calcium-phosphate

## Abstract

Background: To avoid morbidity and limited availability associated with autografts, synthetic calcium phosphate (CaP) ceramics were extensively developed and used as bone filling materials. Controlling their induced-inflammatory response nevertheless remained a major concern. Strontium-containing CaP ceramics were recently demonstrated for impacting cytokines’ secretion pattern of human primary monocytes. The present study focuses on the ability of strontium-containing CaP to control the human primary bone cell production of two major inflammatory and pro-osteoclastogenic mediators, namely MCP-1 and Gro-α, in response to ceramics particles. Methods: This in vitro study was performed using human primary osteoblasts in which their response to ceramics was evaluated by PCR arrays, antibody arrays were used for screening and real-time PCR and ELISA for more focused analyses. Results: Study of mRNA and protein expression highlights that human primary bone cells are able to produce these inflammatory mediators and reveal that the adjunction of CaP in the culture medium leads to their enhanced production. Importantly, the current work determines the down-regulating effect of strontium-substituted CaP on MCP-1 and Gro-α production. Conclusion: Our findings point out a new capability of strontium to modulate human primary bone cells’ communication with the immune system.

## 1. Introduction

Despite much research in the biomaterial field, the gold standard technique to treat large bone defects still consists of autologous bone grafts. Due to the aging of the population, the morbidity, and the limited amount of harvestable autografts, as well as the risks of disease transmission or immunogenic responses associated with allografts, the development of synthetic bioactive materials containing active molecules or ions continues to spread [1]. The development of innovative biomaterials tends to increase the efficiency and the capability of practitioners to treat aged or diseased patients. New strategies have emerged to develop biomaterials leading to faster and better osseointegration. Synthetic calcium phosphate (CaP) ceramics are widely used as bone filling materials as a substitute to auto/allograft thanks to their similarity with the inorganic phase of bone and their osteoconductive properties. Despite their good biocompatibility and their ability to increase the integration of coated implants [2,3,4], CaP materials demonstrate feeble bone cell stimulation and have a tendency to fragment and generate particles which are known to provoke a pro-inflammatory response, which could be harmful and affect long term implant survival [5,6,7,8]. Alternative ceramics have been developed in order to upgrade their bioactive properties [9], to control the inflammatory process, and to enhance their survival time [10,11,12].

So far, researchers have studied the communication between bone forming cells (osteoblasts) and bone resorbing cells (osteoclasts, giant multinucleated cells formed by the merging of precursors deriving from the monocyte lineage) to better understand diseases due to the dysregulation of osteoformation/osteolysis balance [13,14,15,16]. Such diseases could be local (i.e., periodontitis, aseptic loosening…) [16] or systemic (i.e., osteoporosis…) [13] but always involve inflammatory processes. Research work has been mainly focused on osteoclast precursors’ recruitment, activation, and fusion. Nevertheless, the role of osteoblasts was downplayed though it has to be considered. Osteoblasts are actually described as producers of cytokines and chemokines such as IL-6, IL-8, and CCL2 (MCP-1) [17,18,19,20,21] in response to pro-inflammatory cytokines such as tumor necrosis factor α (TNF-α) and IL-1β [21,22,23]. As such, considering osteoblasts as key cells in the control of bone balance seems tempting. This hypothesis is sustained by recent studies that have highlighted the role of MCP-1 in the formation of osteoclasts and Foreign Body Giant Cells [24] and in the recruitment of osteoclast precursors and stimulation of osteoclastogenesis [25,26]. Recent studies have also implicated members of the CXCL family in osteoclast formation [27,28].

In light of these findings, it seems necessary to take cytokine production by bone forming cells into account when developing new biomaterials. Strontium (Sr) is well-known to modify bone balance towards the osteogenic pathway and it has been widely used in treatments for osteoporosis [29]. Strontium has also been demonstrated to be able to enhance pre-osteoblastic cell replication, to stimulate bone formation [30] and to decrease cytokine production [31] and bone resorption [32,33]. Moreover, recent data demonstrated that Sr-substituted CaP particles were able to modulate cytokine production by human monocytes [34]. Strontium ranelate was furthermore shown to inhibit titanium-induced osteolysis by restraining inflammatory osteoclastogenesis due to wear particles in a mice model. This study revealed that strontium ranelate obviously reduced the secretion of tumor necrosis factor-α and interleukin-1β locally synthesized in animals which received titanium particles [31]. Of interest, to our knowledge, no studies focused on the effect of strontium on the expression or synthesis of CCL2 and CXCL1 (Gro-α) chemokines. In the present work, CaP ceramics and CaP biphasic material substituted with five percent of strontium [11] were used to challenge human primary bone cell responses to Ca-P biomaterials. MCP-1 and Gro-α production were specifically assessed and the inflammatory cytokine secretion profile of primary bone cells in response to biomaterial particles was investigated to shed light on putative key pro-inflammatory mediators that could be down-regulated by strontium substitution.

## 2. Results

### 2.1. CCL2 (MCP-1) and CXCL1 (Gro-α) Expression and Synthesis in Basal Conditions

**Figure 1:**
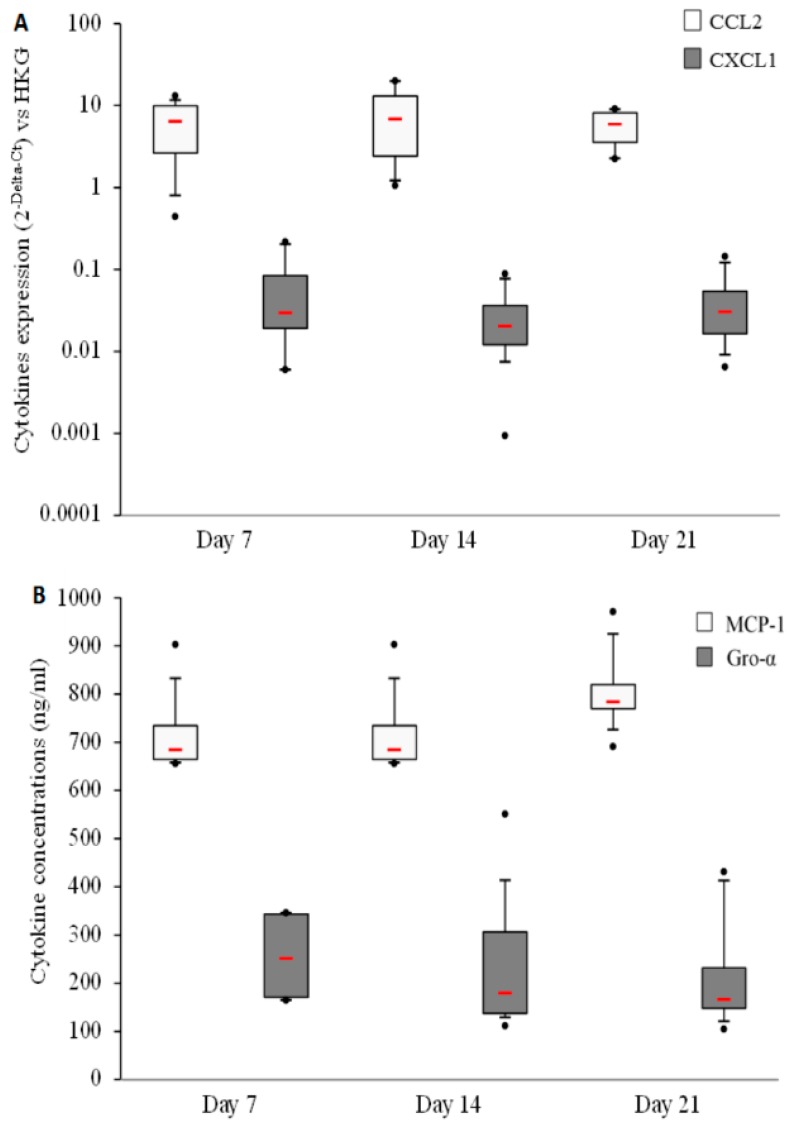
Gro-α and MCP-1 are produced by human primary bone cells in basal culture conditions. (**A**) Human primary bone cells’ cytokine expressions were measured using Real-Time Polymerase Chain Reaction (RT-PCR) after 7, 14, and 21 days of culture. The HPRT1 gene was used as the housekeeping gene (HKG); (**B**) Cytokine concentrations were measured in human primary bone cell culture supernatants after 7, 14, and 21 days. Both experiments were performed on 8 independent donors in duplicate. Red bars represent median values. Black points represent maximum and minimum values. Black bars represent first and ninth deciles and the limits of rectangles represent first and third quartiles.

First, CCL2 (MCP-1) and CXCL1 (Gro-α) expression and synthesis were measured in basal conditions to assess the capability of human primary bone cells to produce both the latter cytokines. The results highlight an important expression (Figure 1A) and production (Figure 1B) of CCL2 (MCP-1) and lower expression and production of CXCL-1 (Gro-α). No differences could be observed between the three culture times (7, 14, and 21 days).

### 2.2. Modulation of CCL2 (MCP-1) and CXCL1 (Gro-α) Expression and Synthesis by Human Primary Bone Cells after CaP Particles Contact

**Figure 2:**
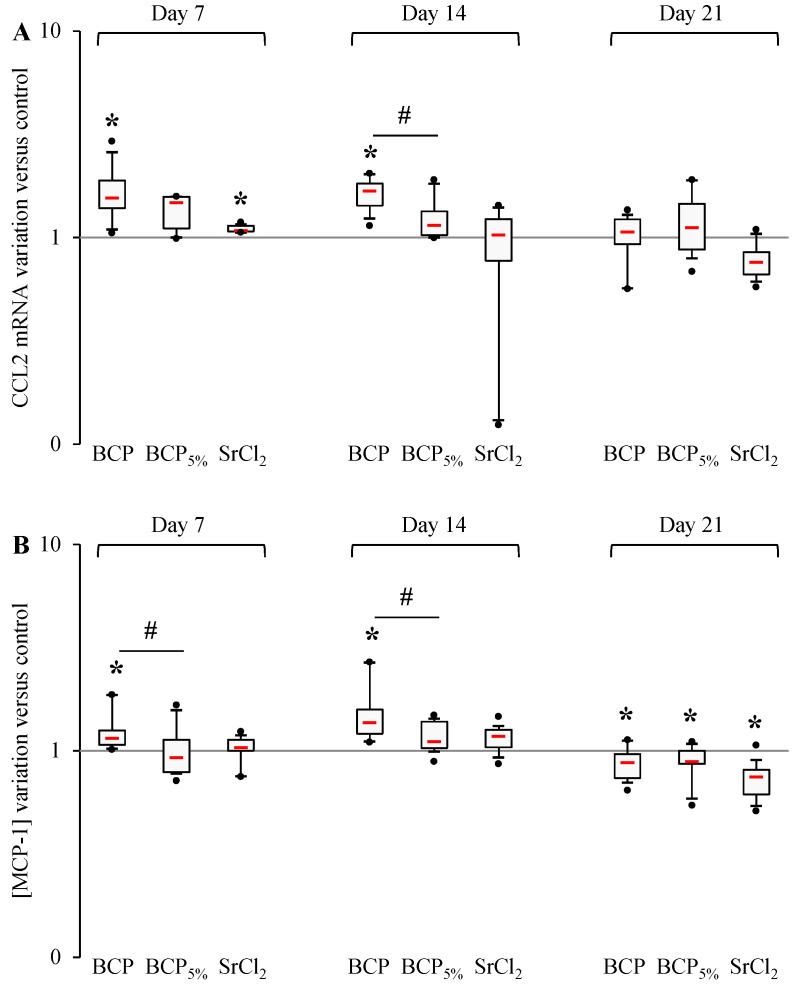
Strontium effect on MCP-1 production by human primary bone cells. (**A**) CCL2 and mRNA expression was evaluated by RT-PCR analysis performed (as described in the Materials and Methods section) after 7, 14 and 21 days of stimulation by BCP, BCP_5%_, and SrCl_2_. Experiments were performed on 5 independent donors in duplicate. Data are shown as specific variation of target genes mRNA using the 2^−ΔΔCt^ method (HPRT1 was used as internal control); (**B**) MCP-1 concentration was measured in cell supernatants after 7, 14, and 21 days of stimulation by BCP, BCP_5%_, and SrCl_2_. * means *p* < 0.05 when compared with control and ^#^ *p* < 0.05 when compared with BCP. Red bars represent median values. Black points represent maximum and minimum values. Black bars represent first and ninth deciles and the limits of white rectangles represents first and third quartiles.

**Figure 3:**
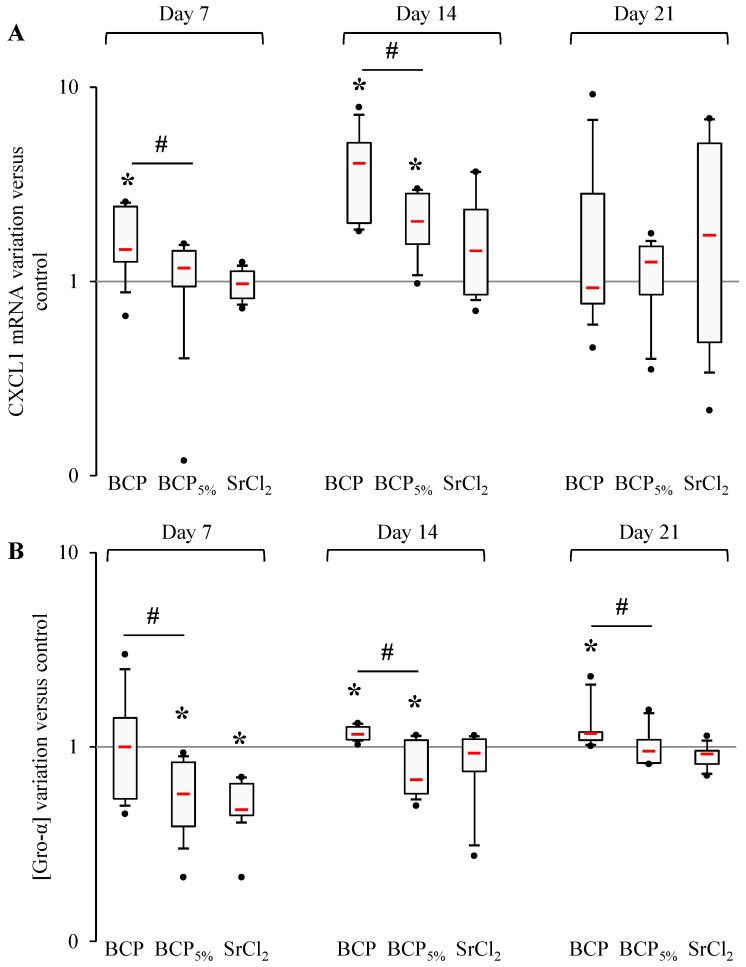
Strontium effect on Gro-α production by human primary bone cells. (**A**) CXCL1 and mRNA production were evaluated by RT-PCR analysis performed (as described in the Materials and Methods section) after 7, 14, and 21 days of stimulation by BCP, BCP_5%_, and SrCl_2_. Experiments were performed on 5 independent donors in duplicate. Data are shown as specific variation of target genes mRNA using the 2^−ΔΔCt^ method (HPRT1 was used as internal control); (**B**) Gro-α concentration was measured in cell supernatants after 7, 14, and 21 days of stimulation by BCP, BCP_5%_, and SrCl_2_. * means *p* < 0.05 when compared with control and ^#^ *p* < 0.05 when compared with BCP. Red bars represent median values. Black points represent maximum and minimum values. Black bars represent first and ninth deciles and the limits of white rectangle represents first and third quartiles.

To highlight the effect of strontium substitution in CaP particles on the expression and synthesis of CCL2 (MCP-1) and CXCL1 (Gro-α), strontium-substituted particles were assessed comparatively with non-substituted ones. Strontium chloride was used as a control. Both studied cytokines revealed a transient increase of expression and synthesis in contact with Biphasic Calcium Phosphate (BCP) during the study (Figure 2 and Figure 3). After 7 days and 14 days of culture with BCP particles, an increase of CCL2 transcription could be observed as well as an increase of MCP-1 secretion, whereas after 21 days both signals decreased. After 7 and 14 days, human primary bone cell exposition to BCP_5%_ did not induce any variation in MCP-1 production (nor at the transcriptional or the translational levels), whereas after 21 days the MCP-1 concentration was decreased in cell culture supernatant without any mRNA variation. Over the same period, SrCl_2_ induced a cellular response similar to BCP_5%_ with a unique difference; a slight increase at the mRNA level after 7 days. Of note, after 7 and 14 days, MCP-1 concentration was significantly lower in BCP_5%_-stimulated cell supernatants in comparison with the BCP condition (Figure 2A,B). After 14 days, BCP particles also induced an increase in CCL2 expression compared to BCP_5%_ conditions.

Regarding Gro-α (Figure 3) after 7 days, BCP particles induced an increased CXCL1 mRNA expression but failed to induce any variations compared to the control condition at the protein level. After 14 days, increased CXCL1 mRNA production and Gro-α concentration were measured when cells were placed with BCP particles, whereas after 21 days only an increase in Gro-α concentration was induced. Under BCP_5%_ stimulation, mRNA stayed at the basal level after 7 days and was increased after 14 days, and BCP_5%_ elicited a decrease in Gro-α concentration after both 7 and 14 days. After 21 days, neither CXCL1 mRNA production nor Gro-α concentration were affected by contact with BCP_5%_ particles. Human primary bone cell treatment with SrCl_2_ did not induce any variations in CXCL1 mRNA expression but elicited a decrease in Gro-α concentration after 7 days. After 14 and 21 days, SrCl_2_ failed to induce any variations both at the mRNA and protein levels. Of importance, CXCL1 mRNA was significantly reduced compared to the BCP condition after 7 and 14 days and the protein level was also decreased by BCP_5%_ condition at all times.

To summarize, we have demonstrated that cells in contact with BCP_5%_ exhibited a lower production of CCL2 and CXCL1 than those exposed to BCP at 7 and 14 days of culture, and SrCl_2_ induced quite the same response pattern as BCP_5%_.

### 2.3. Cytokine Production and the Effect of Biphasic Calcium-Phosphate Particles

**Figure 4:**
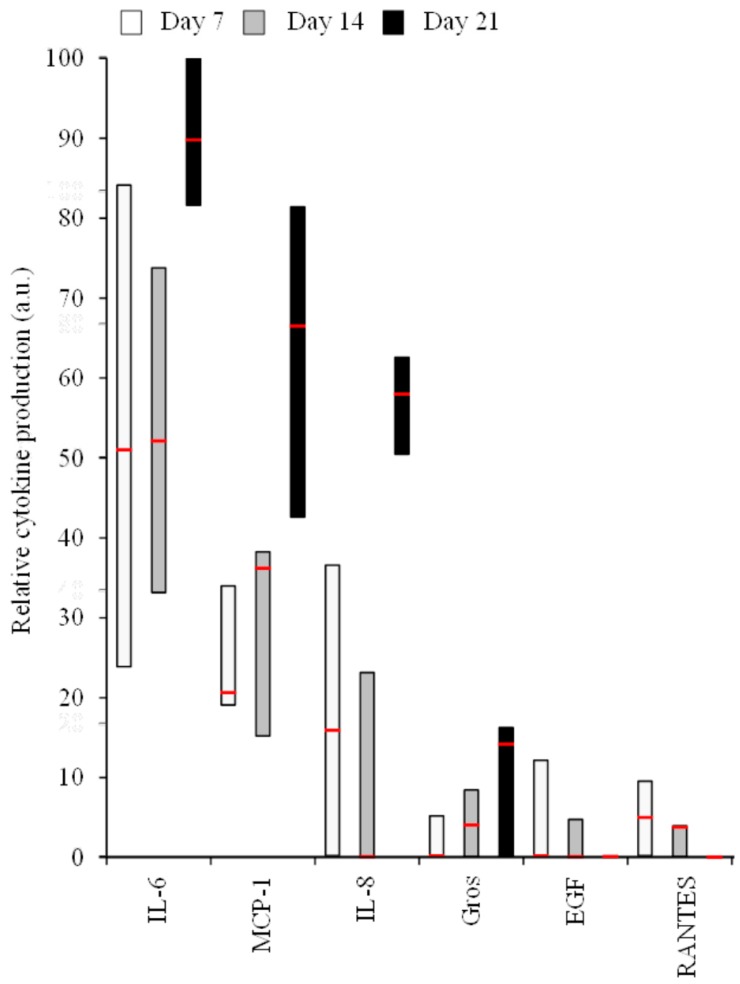
Relative cytokine production in human primary bone cell supernatants in basal culture conditions, measured by antibody arrays. The minimal (bottom box), median (inside box line), and maximal (top box) production are shown from 3 independent donors.

Since interesting results were obtained while studying only two cytokines, we decided to investigate the ability of human primary bone cells to produce a wider range of pro-inflammatory mediators at the protein level in basal conditions (Figure 4), but also in the presence of the BCP particles (Table 1 and Table 2), using antibody array techniques. IL-6, MCP-1, IL-8, Gros, EGF, and RANTES represent the main produced cytokines, and among them three showed high production levels (IL-6, MCP-1, and IL-8) (Table 1). Only IL-6 and MCP-1 were notably detected in all conditions. Others were synthesized by human primary bone cells of one or two of the three initial donors, independently of the studied condition. Finally, GCSF, Gro-α, IL-5, IL-12, IL-13, IFN-γ, MIG, MIP-1δ, TNF-α, and Thrombopoietin were not detected using this technique. We then focused on the six most produced cytokines.

In basal conditions, time dependency could be hypothesized for IL-6, MCP-1, IL-8, and Gros, whereas EGF level remained close to the detection threshold and tends to decrease over time, as observed for RANTES (Figure 4).

In the presence of BCP, a transient increase of cytokine production could be observed for all of the six most produced cytokines after 7 days of interactions (Table 2). Apart from MCP-1, a decrease could be observed on day 14. After 21 days of culture in the presence of BCP, IL-6, MCP-1, IL-8 and Gros productions were minor compared to the control. Conversely, for EGF and RANTES, BCP induced an increased production at day 21.

The adjunction of strontium tended to reduce IL-6 production as revealed by the level of production seen in BCP_5%_ and SrCl_2_ conditions compared to basal and BCP conditions after 7 and 14 days of contact, where strontium was allowed to cut the IL-6 production versus the control only. This difference was also observed in MCP-1 production on day 7 and 14. After an initial increase of IL-8 production on day 7, due to the incorporation of both particles, BCP tended to reduce the production on day 14 and 21. BCP_5%_ tends to restore the IL-8 production to the basal state on day 14 and 21. Production of Gros presented a transient increase of production on day 7 when BCP was employed. This tended to be regulated on days 14 and 21 and soluble or particulate strontium led to an increase of the production of total Gros for all times studied. No clear variation patterns were observed in the presence of strontium for EGF and RANTES.

Our results demonstrated that human primary bone cells are able to secrete some inflammatory mediators at the basal level, and that they are able to modulate this production in response to BCP particles and BCP_5%_ particles.

## 3. Discussion

To our knowledge, this study is the first to evaluate the effect of strontium—especially of BCP containing strontium—on CCL2/MCP-1 and CXCL1/Gro-α expression and production by human primary bone cells. Similar results were obtained when the MCP-1 production was assessed by ELISA or cytokine arrays. Interestingly, Gro-α could not be detected when using cytokine arrays even if total Gros was detected. The detection threshold chosen by the manufacturer could explain this result since those arrays are mainly destined to measure the production of high producing cells from the inflammatory system and since Gro-α is produced in a lower amount than MCP-1. The two most expressed cytokines detected in the present study are IL-6 and MCP-1. In many bone diseases, inflammation is recognized as a key factor which is able to promote and enhance bone destruction [17,35]. Among pro-inflammatory factors acting on bones, TNF-α, IL-6, IL-1β, and the receptor activator of nuclear factor (NF)-κB (RANK)/RANK ligand (RANKL)/osteoprotegrin (OPG) triad [22,35] have been largely described for their tight regulation of mechanisms that govern osteoclast development and activity. Nevertheless, if it is also found that osteoblasts are highly responsive to such immune-derived cytokines, to date, very few studies have tried to assess the ability of primary osteoblasts to express and synthesize cytokines and chemokines despite their central role in communication with the immune system. The present study revealed that human primary bone cells have the capacity to express a wide range of chemokines dedicated to the recruitment and the activation of the monocytes/macrophages (MCPs) and the neutrophils (Gros, IL-8). In the presence of BCP particles, human primary bone cells increased the expression and synthesis of pro-osteoclastic cytokines (Gros, IL-6, IL-8, MCP-1), and some factors which are able to enhance angiogenesis (EGF). They have all been identified to impact the bone balance [36,37,38,39,40]. In addition to the demonstration that the adjunction of BCP in the cell medium tended to increase CXCL1 expression and secretion by human primary bone cells, this work also highlighted that the use of strontium containing BCP allows the reduction of the production of Gro-α and MCP-1. Of importance, MCP-1 is known to be involved in osteoclast differentiation, especially in the cell–cell fusion of monocytic precursors leading to the production of active osteoclasts [25,39,41,42]. It has also been proven that exogenous MCP-1 applied to inflamed bone enhances the recruitment of monocytes which produce factors including (but not limited to) prostaglandins, platelet-derived growth factor, IL-1, and TNF-α, which influence bone metabolism in an osteoclastic way [39,41]. Moreover, works using murine macrophages cultured on Biocoat™ osteologic disks have allowed the identification of a correlation between mineral dissolution activity (due to osteoclastic commitment) and MCP-1 concentration added in the culture medium. Of importance, other researchers have demonstrated such an effect with the same range of cytokine concentrations that were measured in our present study [25,43,44]. In addition, in vivo injection of comparable MCP-1 concentrations into the mice distal femoral canal has induced a systemic recruitment of macrophages [45,46]. Therefore, it can be hypothesized that implantation of BCP may induce an increase of the osteoclast recruitment and differentiation through the modification of the cytokines in the bone micro-environment. The strontium substitution may lead, as previously demonstrated in monocytes [31], to a lower amount of osteoclastogenic cytokine secretion by human primary bone cells in their micro-environment.

Of interest, Gro-α is known to be a potent neutrophil chemoattractant with recognized roles in angiogenesis and inflammation, but it is also considered now as having a local action through the PTHR1, itself being able to stimulate cells of the osteoblastic lineage to release chemokines capable of attracting osteoclast precursors to the bone environment [36]. More recent studies have highlighted that Gro-α and Gro-β are necessary for LPS-induced osteoclast formation [27] and are able to efficiently accelerate osteoclast maturation leading us to consider Gro-α and Gro-β as key cytokines in the bone physiology [28]. In that way, IL-6 tends to present similar modifications of production similar to MCP-1. Strontium tends to reduce the production of IL-6 which is known to be preferentially produced in response to local bone-resorbing agents, and to induce bone resorption both alone and in accordance with other bone-resorbing agents [47]. In addition, IL-6 exhibits a dual role in osteoclastogenesis. On the one hand, IL-6 may directly act on osteoclast progenitors and suppress their differentiation [48,49]; on the other hand, IL-6 is also directly capable of inducing osteoclast formation by a RANKL-independent mechanism [50].

BCP containing strontium presented similar patterns of action on IL-8 than non-strontiated BCP. The addition of IL-8 to cultures of stromal osteoblastic cells is known to stimulate both RANKL mRNA expression and protein production, with no effect on the expression of osteoprotegerin, and to stimulate directly the differentiation of human peripheral blood mononuclear cells into bone-resorbing osteoclasts [40]. Moreover, IL-8 is also considered to be one of the most potent chemoattractant molecules that, among several other functions, guides neutrophils through the tissue matrix until they reach sites of injury [51]. Thanks to these properties, the use of BCP containing strontium could be clinically interesting if employed as biomaterials to ensure the aseptic state of the site of operation. This is strengthened by the decreased production of other CXC (i.e., Gro-α) chemokines. Nevertheless, more studies have to be carried out to determine which CXC cytokine modulation could explain the results obtained with the screening technique. In fact, apart from CXCL1, little is known about the activity of CXCL2 and CXCL3 on bone physiology. EGF and RANTES have also both been implicated in bone remodelling physiology [52]. EGF is able to stimulate the proliferation of preosteoblastic cells as well as to inhibit their further differentiation into osteoblastic cells. This leads to osteoblasts which have a wider capability to migrate into the tissues. Moreover, EGF also has the capability to strongly stimulate osteoclast formation by regulating the expression of OPG and MCP-1 in osteoblastic cells [53]. On the other hand, RANTES is known to be a potent osteoblast chemoattractant and a survival-promoting molecule for these cells [54]. It is also known to potently stimulate the chemotactic recruitment and RANKL-induced differentiation of pre-osteoclasts and to increase the migration of mature osteoclasts [55]. This dual role tends to lead to an increase in bone remodelling. The results presented here tend to confirm that human primary bone cells are involved in the host immune response to particles. BCP particles have been demonstrated to be able to increase the cytokine production by human primary bone cells and Sr-substitution of BCP may be a promising approach to reduce such an over-production. This could impact bone remodelling as BCP_5%_ can modulate both Gro-α and MCP-1 secretion. In vivo studies are needed to confirm those parameters and to test such biomaterials in a more complex system.

## 4. Materials and Methods

### 4.1. Reagents

Dulbecco’s phosphate-buffered saline 1X (DPBS), Trypsin, Ethylenediaminetetraacetic acid (EDTA), Penicillin, Streptomycin, Dulbecco’s Modified-Eagle Medium (DMEM) + Glutamax, collagenase II, and foetal calf serum (FCS) were purchased from Invitrogen/Gibco (Life Technologies, Carlsbad, CA, USA). SrCl_2_, Ca(NO_3_)_2_·4H_2_O, Sr(NO_3_)_2_·4H_2_O, and P_2_O_5_ for the Biphasic Calcium Phosphate (BCP) and 5% strontium-substituted BCP (BCP_5%_) powder syntheses were from Sigma Aldrich Chemicals (St. Louis, MO, USA) and Avocado Research Chemicals (London, UK), respectively. The RNeasy Micro Kit and RNase-Free DNase Set were from Qiagen. Power SYBR^®^ Green PCR Master Mix and High capacity reverse transcription kit were from Applied Biosystems (Life Technologies).

### 4.2. Cells

Eight femoral heads of bone explants were obtained from the Orthopaedic and Traumatology department of the University Hospital of Reims, France. Samples were collected after written informed consent from the donors. The retrieved explants were cut into small pieces, extensively washed in phosphate-buffered saline, digested in a solution of 0.5X trypsin/EDTA, and then in collagenase II (140 mg·mL^−1^). Fragments were then incubated in culture flasks containing Dulbecco’s modified Eagle medium supplemented with 20% fetal calf serum, 1% antibiotic solution containing penicillin (10,000 U·mL^−1^), and streptomycin sulphate (10,000 µg·mL^−1^) at 37 °C in a 5% CO_2_ humidified atmosphere. In all experiments, third passage cultures were used. Bone cells were seeded at a density of 1 × 105 cells per well in 6 well culture plates (BD Falcon). They were cultured in DMEM supplemented with 10% FCS, 0.5% antibiotic solution penicillin (10,000 IU·mL^−1^), and streptomycin sulphate (10,000 µg·mL^−1^) and incubated at 37 °C in a 5% CO_2_ humidified atmosphere. BCP, BCP_5%_, and SrCl_2_ were added to the culture medium when the cells were confluent. In the present work, the Surface Area Ratio method (SAR—defined as surface area of material/surface area of cells) was used to choose the amount of BCP and BCP_5%_ incorporated in human primary bone cell cultures Based on our previous data, we chose a SAR equal to 1 for both materials in our experiments [9] and we added a control condition containing 20 µM of SrCl_2_ at the initial time to have an insight on the strontium effect independently of the presence of particles [33]. Supernatants were then collected after 7, 14, and 21 days of culture for ELISA and antibody arrays and cells were harvested for PCR arrays and RT-qPCR studies.

### 4.3. Biomaterials

The production and characterization of pure and strontium-substituted BCP powders have been reported previously [9,33]. The particle size range was from 1 to 100 µm (the mean particle size as determined by laser granulometry was around 30 µm, with significant polydispersity) [9,33]. Briefly, the sol–gel route previously proposed by the authors was used. To produce 2 g of pure HAp powder, 4.7 g of Ca(NO_3_)_2_·4H_2_O (Aldrich) and 0.84 g of P_2_O_5_ (Avocado Research chemicals) were dissolved in ethanol under stirring and refluxed at 85 °C for 24 h. Then, this solution was kept at 55 °C for 24 h, to obtain a white consistent gel and was further heated at 80 °C for 10 h to obtain a white powder. Finally, the powder was heated at 1100 °C for 15 h. To prepare Sr-substituted hydroxyapatite, the required amount of Sr(NO_3_)_2_ (Aldrich) was added to the solution. In the presented work, particles were tested and found to be endotoxin-free using the E-toxate kit from Sigma-Aldrich (Saint-Quentin-Fallavier, Isère, France).

### 4.4. RNA Purification and Reverse-Transcription

Total RNAs were extracted and cleaned up with RNeasy Micro Kit. Residual genomic DNA was removed by an on-column DNAse digestion step using a RNase-Free DNase Set. mRNA quantitation was realized on a Qubit quantitation platform (Invitrogen, Carlsbad, CA, USA) and RNA quality was checked by gel electrophoresis. Reverse transcription directly followed the use of the high capacity reverse transcription kit (Applied Biosystems, Carlsbad, CA, USA) on 1 µg of total RNAs.

### 4.5. Real Time PCR Primer Design and Efficiency Determination

Real time PCR experiments were realized with an ABI7000 thermal cycler (Applied Biosystems). A calibrator was made by pooling a small amount (5 µL) of all cDNA obtained. Primers were designed for CCL2 (MCP-1) and CXCL1 (Gro-α) (Table 3). HPRT1 was used as an internal control. For each Primer couple the specificity and efficiency were determined by testing them against this calibrator. Results were calculated using the ΔCt method (data were expressed as the difference of Ct between the gene studied and a housekeeping gene (HPRT1)). Data presenting more than one peak in their melting curves or Cycle threshold over 35 were not considered.

### 4.6. Enzyme-Linked Immunosorbent Assay (ELISA)

MCP-1 and Gro-α concentrations were measured in culture supernatants coming from 8 independent donors using the DuoSet ELISA kit (R & D Systems), following the manufacturer’s instructions. Controls included supernatant from non-stimulated cells and medium alone.

### 4.7. Antibody Cytokine Arrays

Cytokine antibody arrays (“Human Cytokine Array C3”, Raybiotech, Norcross, GA, USA) were performed after 7, 14, and 21 days of culture according to the manufacturer’s instructions. Briefly, 10-fold diluted culture supernatants coming from 3 independent donors or medium alone were incubated on antibody-coated membranes before detection with a streptavidin-horseradish peroxidase biotinylated-antibody complex and chemiluminescence detection on X-ray films for 2 min. The integrated optical density (IOD) of each dot was measured on digitalized autoradiograms with the ImageJ software (1.50i, National Institutes of Health, Bethesda, MD, USA) after subtraction of the non-specific signal. Each value corresponds to the ratio between the dot studied and the internal positive control (expressed as the average of six internal control dots). Values under the detection threshold (determined by the maximum value of the negative internal control dots) were considered as non-achievable (NA). Data were calculated as the average level of secretion (arbitrary units) for each mediator normalized with internal positive controls and internal negative control.

### 4.8. Statistics

The significance of the results was assessed with exact nonparametric and stratified Wilcoxon Mann Whitney tests (StatXact 7.0, Cytel Inc., Cambridge, MD, USA). We used nonparametric statistics owing to a lack of normal distribution of the assessed variables. Stratification allowed the impact of donor variability to be taken into account. Differences were considered significant at *p* < 0.05.

## Figures and Tables

**Table 1:** Cytokine array inflammatory mediator screening. Values express the average (*n* = 3) level of secretion (arbitrary units) for each mediator normalized with internal positive controls and an internal negative control. Frequency represents the number of conditions where cytokines could be detected.

Level of Production	Cytokines	Frequency of Detection	Relative Mean Level Detected
**Produced by all donors in all conditions**	IL-6	36	66
	MCP-1	36	47
**Produced by a majority of donors in a majority of conditions**	IL-8	26	40
	GRO	19	12
	EGF	19	9
	RANTES	18	8
**Produced by a minority of donors in a minority of conditions**	IL-7	15	9
	ENA-78	11	6
	TARC	10	6
	IL-3	9	6
	Leptin	9	5
	IGF-1	8	5
	GM-CSF	7	5
	I-309	7	5
	IL-1α	7	4
	MCSF	6	4
	SCF	6	5
	TGF-β1	6	4
	TNF-α	6	5
	Oncostatin M	6	4
	SDF-1	5	5
	Angiogenin	5	7
	PDGF BB	5	4
	IL-10	2	4
	MCP-2	2	5
	MCP-3	2	4
	VEGF	2	4
	IL-1β	1	4
	IL-2	1	5
	IL-4	1	4
	IL-15	1	4
	MDC	1	4

**Table 2:** Average values of cytokine production in human primary bone cell culture supernatants in BCP, BCP_5%_, and SrCl2-stimulated conditions measured by antibody arrays. NA means not achievable.

Cytokines	Day 7				Day 14				Day 21			
	Basal	BCP	BCP_5%_	SrCl_2_	Basal	BCP	BCP_5%_	SrCl_2_	Basal	BCP	BCP_5%_	SrCl_2_
**IL-6**	63.46	68.80	57.50	43.65	63.63	61.75	52.24	59.61	91.41	69.82	78.74	85.96
**MCP-1**	29.31	53.58	42.87	30.02	35.85	50.81	47.32	37.59	63.52	55.65	62.41	69.94
**IL-8**	20.94	28.16	37.63	29.02	9.32	6.42	9.97	8.73	57.01	41.55	43.17	56.64
**GROs**	2.10	4.03	6.68	3.47	5.02	1.62	6.16	7.16	10.17	9.79	12.20	10.26
**EGF**	4.87	9.35	10.93	8.27	5.57	1.83	7.19	2.46	NA	8.00	7.19	2.46
**RANTES**	5.75	7.03	7.72	7.72	3.12	1.92	2.01	2.16	NA	5.15	4.79	6.52

**Table 3:** Nucleotide sequences of primers used for RT-PCR and reaction efficiency for each Primer couple.

Targeted mRNA	Sense Primer (5′-3′)	Antisense Primer (5′-3′)	Efficiency
CXCL1	TCCTGCATCCCCCATAGTTA	CTTCAGGAACAGCCACCAGT	1.97
CCL2	AGTCTCTGCCGCCCTTCT	GTGACTGGGGCATTGATTG	1.98
HPRT1	TGACCTTGATTTATTTTGCATACC	CGAGCAAGACGTTCAGTCCT	1.99
